# Development and Congenital Anomalies of the Pancreas

**DOI:** 10.1155/2011/351217

**Published:** 2011-05-14

**Authors:** Hiroyuki Tadokoro, Masaru Takase, Bunsei Nobukawa

**Affiliations:** ^1^Department of Gastroenterology, Asakadai Central Hospital, 1-8-10 Nishibenzai, Asaka, Saitama 351-2055, Japan; ^2^Department of Human Pathology, Juntendo University, 2-1-1 Hongo, Bunkyo-ku, Tokyo 113-8421, Japan

## Abstract

Understanding how the pancreas develops is essential to understand the pathogenesis of congenital pancreatic anomalies. Recent studies have shown the advantages of investigating the development of frogs, mice, and chickens for understanding early embryonic development of the pancreas and congenital anomalies, such as choledochal cysts, anomalous pancreaticobiliary junction, annular pancreas, and pancreas divisum. These anomalies arise from failure of complete rotation and fusion during embryogenesis. There are many theories in the etiology of congenital anomalies of the pancreas. We review pancreas development in humans and other vertebrates. In addition, we attempt to clarify how developmental failure is related to congenital pancreatic anomalies.

## 1. Introduction

In the 19th century, early embryonic development of the pancreas in mammals and other vertebrates was investigated. Many histological studies of human and other mammalian embryos have confirmed that the ventral pancreatic anlage occurs in a paired condition [1–7]. It is believed that the ventral pancreatic anlage is initially paired, with the left lobe subsequently disappearing during development [1, 2, 8, 9]. Recent research has examined pancreas development using animal models. It has become clear that early pancreas development in humans closely resembles that of mice and frogs [9, 10], whereas in chickens and frogs, the left ventral anlage persists, and the two ventral buds fuse together and become part of the mature organ [10, 11]. It is considered that mammals, birds, reptiles, and amphibians have similar development [12]. 

There are various types of congenital anomalies of the pancreas in humans. Choledochal cysts are anomalies of the bile ducts, which manifest as dilatation of intra- and extrabiliary trees. Choledochal cysts of the pancreas have an abundance of pancreatic tissue in the head of the organ. Anomalous pancreaticobiliary junction (APBJ) is a congenital anomaly in which the pancreatic and biliary ducts join outside the duodenal wall and form an abnormally long common channel [13]. This anomaly is closely related with choledochal cysts, because >90% of cysts are complicated with APBJ. However, APBJ without biliary dilatation (non dilatation-type APBJ) is another congenital anomaly of the pancreas, which has an abnormally shaped pancreatic head [8, 14]. Annular pancreas is a well-known congenital anomaly in which pancreatic tissue surrounds the second portion of the duodenum [15]. Two main theories for formation of annular pancreas have been proposed. One is that the left ventral anlage persists and that the right ventral anlage does not rotate around the duodenum. The other theory is that the right ventral anlage stretches and encircles the duodenum. Pancreas divisum is a congenital anomaly in which ventral and dorsal pancreatic ducts do not fuse together. These congenital anomalies are responsible for embryonic developmental failure. Recognition of normal development of the pancreas in mammals and other species helps us to understand congenital anomalies in humans. The aim of this paper is to review normal development of the pancreas and how this relates to human congenital anomalies.

## 2. Embryonic Development of the Pancreas and Biliary Tree

Ventral (caudal) and dorsal (cranial) outpouchings develop at the junction of the foregut and midgut during the fourth week of gestation. The dorsal diverticulum forms the dorsal portion of the pancreas, and the ventral diverticulum forms the liver, gallbladder, bile ducts, and ventral pancreas. As the foregut elongates, the developing ventral pancreas, gallbladder, and bile duct rotate clockwise posterior to the duodenum and join the dorsal pancreas in the retroperitoneum. The ventral pancreatic duct and the common bile duct (CBD) are linked by their embryonic origins, which results in the adult configuration of their common entrance into the duodenum at the major papilla [16]. The ventral pancreatic bud fuses with the dorsal bud at approximately the seventh week of gestation. During the eighth week of gestation, the remaining portion of the ventral diverticulum separates into the pars cystica and pars hepatica [17]. The pars cystica forms the cystic duct and gallbladder. The pars hepatica branches to form the two major lobes of the liver. The proliferation is followed by vacuolation, and the coalescence of the ensuing lacunae produces a tubular biliary duct system. The pars cystica vacuolates and expands, and the stalk becomes the cystic duct. This structure is initially hollow, then solid (by proliferation of epithelial lining), and recanalization occurs by vacuolation of this expanded epithelium [18].

**Figure 1:**
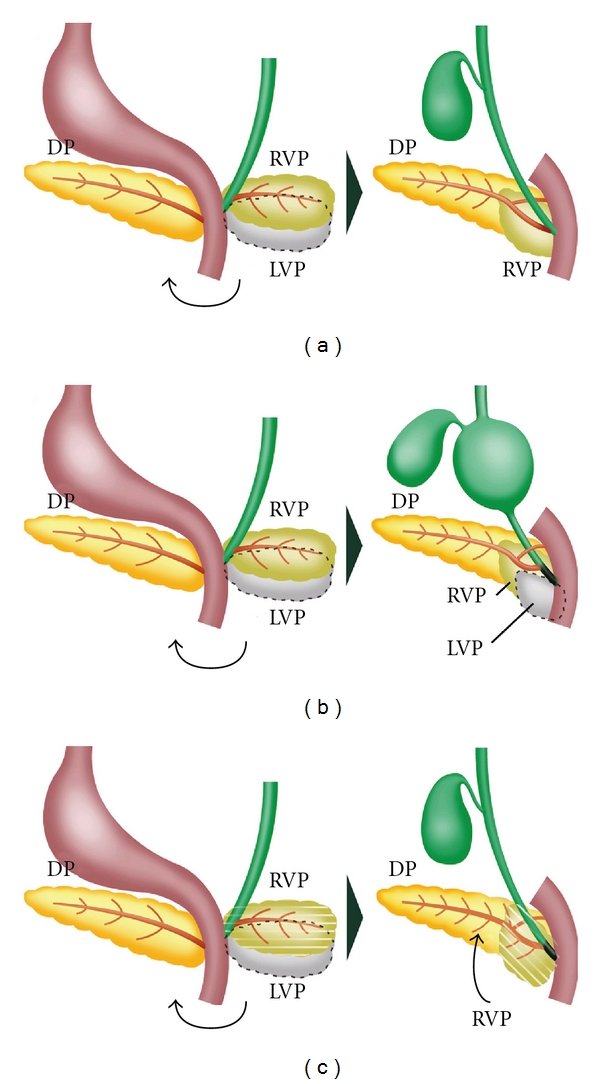
(a) Normal development of the pancreas. The ventral pancreatic anlage is initially paired, with the left lobe subsequently disappearing during development. The ventral pancreatic anlage fuses side by side with the dorsal anlage. (b) Choledochal cysts can occur when the left ventral anlage persists and disturbs normal bile duct recanalization. (c) Non dilation-type APBJ occurs when the ventral anlage fuses with the dorsal anlage in an oblique position. RVP, right ventral pancreatic anlage; LVP, left ventral pancreatic anlage; DP, dorsal pancreatic anlage.

Early development of the pancreas has been examined in human, pig, sheep, and other vertebrate embryos. In the early 19th century, it was investigated whether the ventral pancreatic bud occurs in a single or paired condition in humans and other vertebrate embryos [2], and it was discovered that the ventral anlage is paired in human embryos [2–7]. The ventral pancreatic bud is paired in the sheep embryo [19, 20]. Lewis has discovered that the ventral pancreatic bud appears to be a paired organ at first and that the left ventral pancreatic bud degenerates in pig embryos [1]. A pair of ventral pancreatic anlagen have been observed in rabbit, rat, guinea pig, and cat embryos [21]. Recent studies have demonstrated that early pancreas development in humans closely resembles that of mice and frogs [9, 10]. In mammals, it is considered that the pancreas is a single endodermal organ that is embryologically derived from one dorsal and two ventral anlagen [1, 2, 8, 9] (Figure 1(a)). However, in chickens and frogs, the left ventral anlage persists, and the two ventral and dorsal anlagen fuse to form a discrete pancreas [11, 12].

Descriptions of development of the dorsal pancreas are far fewer in number than those of the development of the ventral pancreas. Lewis has described the dorsal pancreatic bud as appearing in a paired condition as well as the ventral pancreas [1]. A pair of dorsal pancreases has been described in sheep embryos [19]. However, this has not been found in humans. Whether the dorsal pancreas occurs in a single or paired condition in humans is unclear.

The ventral and dorsal pancreases can be distinguished by examining the lobular structure and immunohistochemical staining for pancreatic polypeptide (PP). PP cells are localized to the area that is derived from the ventral anlage [22–27]. The ventral pancreas is composed of smaller and more tightly packed lobules with PP-rich islets, whereas the dorsal pancreas is composed of larger lobules with PP-poor islets. The ventral pancreatic bud fuses side by side with the dorsal bud under normal conditions [22, 28, 29]. The dorsal pancreatic bud forms the upper head, body, and tail of the pancreas. The ventral bud forms the inferior head and uncinate process. When both pancreatic buds fuse, the pancreatic duct system starts to establish. The main pancreatic duct forms from the ventral pancreatic duct in the head and the distal part of the dorsal pancreatic duct in the body and tail. The accessory pancreatic duct forms from the dorsal pancreatic duct embryologically. The accessory pancreatic duct (dorsal pancreatic duct) joins the main pancreatic duct (ventral pancreatic duct) at a site 1-2 cm proximal to the ventral pancreatic duct or at the distal end of the ventral pancreatic duct [30, 31]. 

## 3. Choledochal Cysts

Choledochal cysts are a well-known anomaly that appears as dilatation of extra- or intrabiliary trees. Choledochal cysts have been classified into five subtypes radiologically by Todani et al. [32], which is a modification of the Alonso-Lej classification [33]. Choledochal cysts, which are rare and more common in female than male patients, occur in approximately 1 : 100,000–150,000 live births in Western countries [34]. Choledochal cysts are much more prevalent in Asia than in Western countries. Approximately 33%–50% of reported cases come from Japan, where the frequency in some studies has approached one case per 1000 population [35].

Type I cysts consist of fusiform dilatation of the extrahepatic bile duct; this is the most common type and represents nearly 78% of cases. Type II cysts are a diverticulum of the CBD. Type III cysts are also called choledochoceles, which show dilatation of the intraduodenal portion of the CBD. Type IV cysts have two types, with IV-A demonstrating multiple intra- and extrahepatic cysts, and IVb demonstrating only multiple extrahepatic biliary dilatations. The type IV cyst is the second most common type in adults, and represents 10%–15% of adult cases [36]. The type V cyst, also known as Caroli's disease, is a cystic dilatation of the intrahepatic biliary system [37]. Diverticulum of the extrahepatic bile duct (type II), choledochocele (type III), and Caroli disease (type V) are not associated with type I and IV-A choledochal cysts from clinical and embryological standpoints. Embryologically, type I and IV-A cysts seem to belong to a different category from other cysts [38].

**Figure 2:**
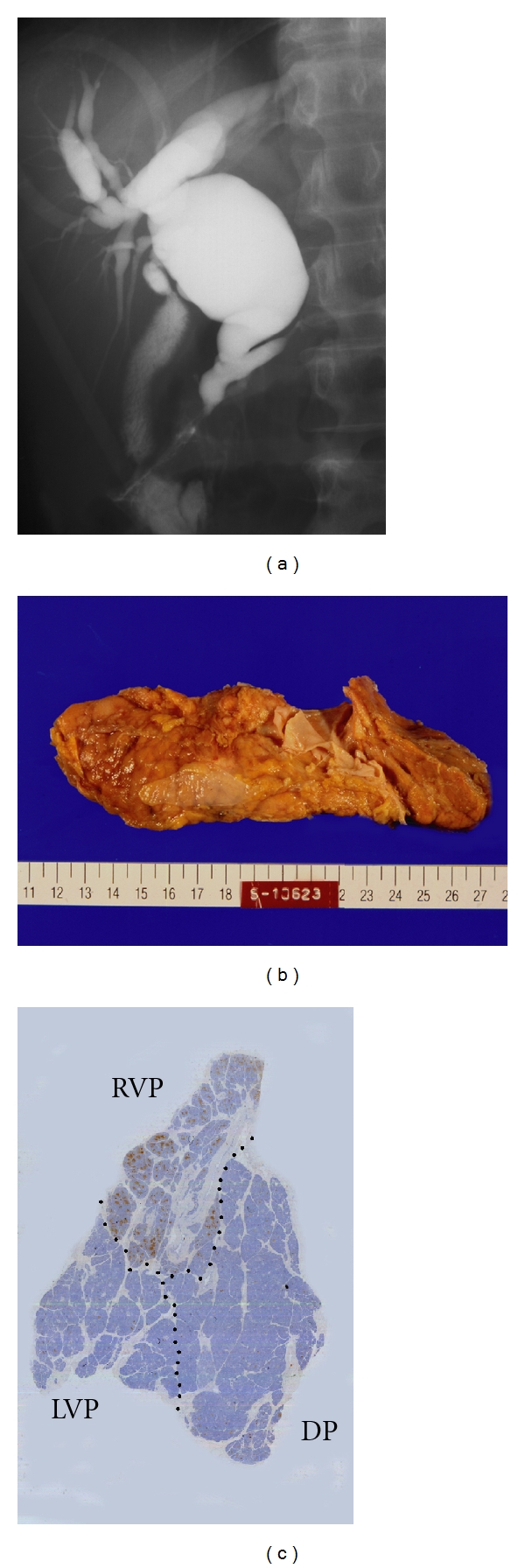
Choledochal cysts (a) ERCP showing the long common channel and dilatation of intra- and extrabiliary ducts. (b) Macroscopic view showing a huge head with abundant pancreatic tissue in the dorsoventral direction. (c) Immunohistochemical staining of pancreatic polypeptide (PP). Distinction between the ventral and dorsal pancreas was done based on immunohistochemistry for PP and the lobular structure. The ventral pancreas was divided into a PP-rich portion and a PP-poor portion immunohistochemically. RVP, right ventral pancreas; LVP, left ventral pancreas; DP, dorsal pancreas.

Type I and IV-A cysts are the most common types and account for nearly 90% of cases. APBJ is seen in >90% of patients with type I and IV-A choledochal cysts [35]. The pancreas with type I and IV-A choledochal cysts has been demonstrated as an anatomical anomaly of the pancreas (Figures 2(a) and 2(b)). The head of the pancreas has abundant pancreatic tissue. Immunohistochemically, the ventral pancreas can be divided into PP-rich and PP-poor lobes. The former are believed to be derived from the right ventral anlage and the latter from the left ventral anlage (Figure 2(c)). In chickens and frogs, the left ventral anlage persists and becomes a mature organ [17, 18]. 

When the ventral and dorsal pancreatic buds fuse, the bile ducts are in solid stage [23, 39]. Recanalization of the bile duct might be delayed by the presence of the left ventral pancreatic anlage. Recanalization of the CBD starts at the middle portion of the duct and extends into the proximal and distal portions. When the proximal and distal sides of the CBD are in a solid state, aberrant recanalization might occur in the middle portion of the duct. Failure of recanalization of the CBD during the solid stage of development leads to dilatation of its middle portion and stenosis of the proximal portion. Choledochal cysts might be caused by the persistence of the left ventral pancreatic bud [8] (Figure 1(b)). 

## 4. APBJ without Dilatation of Bile Ducts (Non Dilated Type-APBJ)

APBJ is a rare congenital anomaly in which the pancreatic and biliary ducts join outside the duodenal wall [13]. APBJ is diagnosed when the pancreatic duct joins the bile ducts 1-2 cm proximal to the sphincter of Oddi [24–27, 40]. The incidence of APBJ has been reported to be 1.5–3.0% in patients who are undergoing endoscopic retrograde cholangiopancreatography (ERCP) for various reasons [24, 41, 42]. It is well known that APBJ is commonly associated with congenital bile duct dilatation and carcinoma of the bile duct and gallbladder. The reason for biliary carcinogenesis in such patients has been ascribed to reflux and stasis of bile mixed with pancreatic juice in the dilated bile duct and gallbladder [43, 44]. The incidence of gallbladder carcinoma and biliary tract cancer in APBJ without bile duct dilatation is 36.1% and 4.0%, respectively, according to the register of the Japanese Study Group on Pancreaticobiliary Maljunction over the past 10 years [45]. 

APBJ is classified into two groups, with or without bile duct dilatation, and is seen in >90% of patients with types I and IV-A choledochal cysts [35]. The etiology of APBJ has been proposed by some authors. Matsumoto et al. have suggested that APBJ is caused by embryonic disarrangement of the distal bile duct and the ventral pancreatic ducts [46]. The pancreaticobiliary ductal junction lies outside the duodenal wall in early fetal life, and the junction comes to lie within the wall during development. Wong and Lister have speculated that the arrest of this migration or the failure of ampullary involution is the cause of APBJ with or without choledochal cysts [47]. 

**Figure 3:**
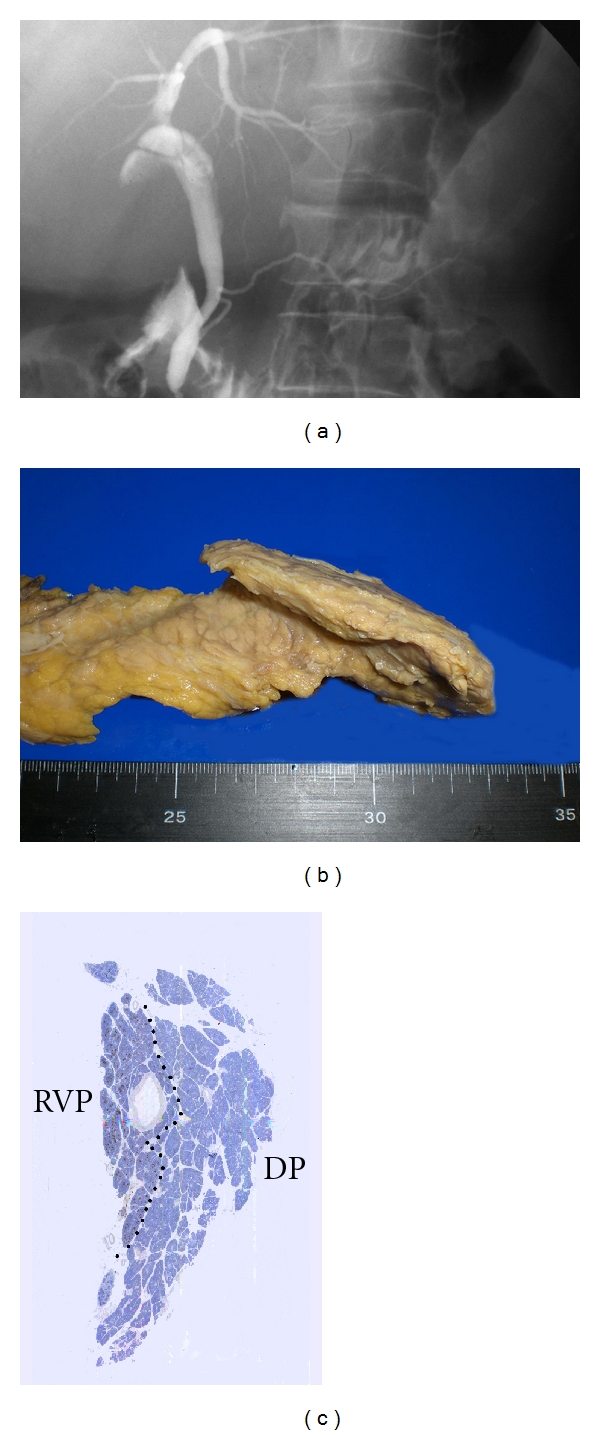
Non dilatation-type APBJ (a) ERCP showing the long common channel without dilatation of bile ducts. (b) Macroscopic view showing abnormal shape of the head. (c) The PP-rich portion (ventral pancreas) was situated obliquely dorsal to the PP-poor portion (dorsal pancreas). RVP, right ventral pancreas; DP, dorsal pancreas. Published permission for Pathology International.

Under normal conditions, the ventral bud fuses side by side with the dorsal bud. Histological and immunohistochemical studies have shown that the ventral pancreas fuses with the dorsal pancreas in an oblique position in non dilatation-type APBJ [8, 14] (Figure 3). As the developing pancreatic duct fuses with the developing bile duct in an oblique position, a long common channel is formed in non dilatation-type APBJ. Non dilatation-type APBJ might be caused by abnormal fusion between the ventral and dorsal anlagen (Figure 1(c)).

## 5. Annular Pancreas

Annular pancreas is a rare congenital anomaly in which a ring of pancreatic tissue surrounds the duodenum. It is estimated that it occur in one of every 12,000–15,000 live births [48]. The annular pancreatic tissue forms a complete (25%) or partial (75%) ring around the descending duodenum [49, 50]. The incidence of annular pancreas has been reported to be 0.005%–0.015% in autopsy cases in adults [51]. It is frequently associated with other congenital abnormalities such as esophageal atresia, imperforate anus, congenital heart disease, malrotation of the midgut, and Down syndrome.

**Figure 4:**
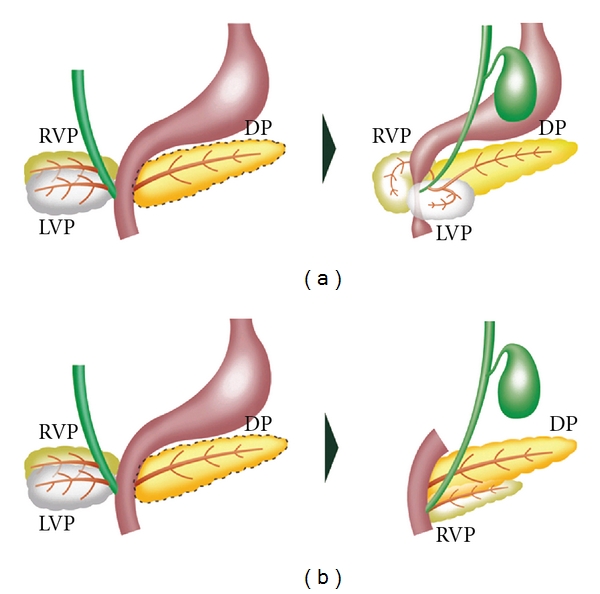
(a) Annular pancreas might be formed when the left ventral pancreatic anlage persists, and the right ventral pancreatic anlage does not rotate around the duodenum. The two ventral anlagen encircle the duodenum. (b) Pancreas divisum. After the left ventral anlage disappears, the right ventral anlage rotates around the duodenum and fuses the dorsal anlage. The ventral and dorsal pancreatic ducts fail to fuse together. RVP, right ventral pancreatic anlage; LVP, left ventral pancreatic anlage; DP, dorsal pancreatic anlage.

There are two main hypotheses to explain pathogenesis of annular pancreas. One is that the tip of the right ventral bud adheres to the duodenal wall and stretches to form a ring during normal rotation, as proposed by Lecco [52]. The other hypothesis is that the left ventral bud persists, which develops to complete a circle of pancreatic tissue around the duodenum, as proposed by Baldwin [53]. Some pathologists support Lecco's hypothesis, because annular pancreatic tissue is composed of PP-rich islets, which is believed to be derived from the right ventral anlage [54, 55]. Although several classifications of annular pancreas have been proposed [56–58], either theory could explain all types of the anomaly [50, 59]. Whether ventral pancreatic bud occurs in a single or paired condition is of importance in the formation of annular pancreas [60, 61]. Annular pancreas is formed by the two ventral and one dorsal pancreases in pigs [1]. The normal pancreas is formed by fusion between two ventral and one dorsal pancreases in chickens and frogs [11, 12]. Therefore, annular pancreas might be formed by persistence of the left ventral bud in the human embryo, when considering the development of the pancreas in other species (Figure 4(a)).

## 6. Pancreas Divisum

Pancreas divisum is the most common congenital anomaly of the pancreas. The ventral and dorsal ducts fail to fuse together, resulting in pancreas divisum [56] (Figure 4(b)). The body, tail, and part of the head of the pancreas (dorsal pancreas) drain through Santorini's duct into the minor papilla, while another part of the head (ventral pancreas) drains through Wirsung's duct into major papilla. This anomaly is found with an incidence of 3%–7% in patients who are undergoing ERCP and in approximately 9% of autopsy cases [16]. The cause of this anomaly is unknown. A short and rudimentary ventral duct in pancreas divisum is thought to be caused by hypoplasia of the ventral pancreas. 

ERCP is regarded as the most definitive and reliable diagnostic method for revealing pancreas divisum. However, ERCP itself sometimes can induce pancreatitis. Magnetic resonance cholangiopancreatography is a non invasive and accurate method in the diagnosis of pancreas divisum. The clinical relevance of pancreas divisum remains controversial. 

Most patients with pancreas divisum are asymptomatic [57–59]. A relative obstruction to pancreatic exocrine secretory flow through the duct of Santorini and minor papilla could result in pancreatitis in a small number of patients with pancreas divisum [62, 63]. 

Endoscopic stenting and sphincterotomy of the minor papilla are feasible and might be effective in some patients with pancreas divisum [64]. 

## 7. Conclusion

Embryonic development of the pancreas in various species offer suggestions about the development and congenital anomalies of the pancreas in humans. 

Understanding how the pancreas develops is essential to understand the pathogenesis of congenital pancreatic diseases.
